# Exonic Variants in Aging-Related Genes Are Predictive of Phenotypic Aging Status

**DOI:** 10.3389/fgene.2019.01277

**Published:** 2019-12-19

**Authors:** Megan E. Breitbach, Susan Greenspan, Neil M. Resnick, Subashan Perera, Aditi U. Gurkar, Devin Absher, Arthur S. Levine

**Affiliations:** ^1^HudsonAlpha Institute for Biotechnology, Hunstville, AL, United States; ^2^Department of Biotechnology Science and Engineering, University of Alabama in Huntsville, Hunstville, AL, United States; ^3^Division of Geriatric Medicine, Department of Medicine, University of Pittsburgh School of Medicine, Pittsburgh, PA, United States; ^4^Institute on Aging of UPMC, University of Pittsburgh School of Medicine, Pittsburgh, PA, United States; ^5^Department of Biostatistics, University of Pittsburgh Graduate School of Public Health, Pittsburgh, PA, United States; ^6^Department of Microbiology and Molecular Genetics, University of Pittsburgh School of Medicine, Pittsburgh, PA, United States; ^7^UPMC Hillman Cancer Center, Pittsburgh, PA, United States

**Keywords:** machine learning, aging, genetics, bioinformatics, sequencing

## Abstract

**Background:** Recent studies investigating longevity have revealed very few convincing genetic associations with increased lifespan. This is, in part, due to the complexity of biological aging, as well as the limited power of genome-wide association studies, which assay common single nucleotide polymorphisms (SNPs) and require several thousand subjects to achieve statistical significance. To overcome such barriers, we performed comprehensive DNA sequencing of a panel of 20 genes previously associated with phenotypic aging in a cohort of 200 individuals, half of whom were clinically defined by an “early aging” phenotype, and half of whom were clinically defined by a “late aging” phenotype based on age (65–75 years) and the ability to walk up a flight of stairs or walk for 15 min without resting. A validation cohort of 511 late agers was used to verify our results.

**Results:** We found early agers were not enriched for more total variants in these 20 aging-related genes than late agers. Using machine learning methods, we identified the most predictive model of aging status, both in our discovery and validation cohorts, to be a random forest model incorporating damaging exon variants [Combined Annotation-Dependent Depletion (CADD) > 15]. The most heavily weighted variants in the model were within *poly(ADP-ribose) polymerase 1* (*PARP1*) *and excision repair cross complementation group 5* (*ERCC5*), both of which are involved in a canonical aging pathway, DNA damage repair.

**Conclusion:** Overall, this study implemented a framework to apply machine learning to identify sequencing variants associated with complex phenotypes such as aging. While the small sample size making up our cohort inhibits our ability to make definitive conclusions about the ability of these genes to accurately predict aging, this study offers a unique method for exploring polygenic associations with complex phenotypes.

## Introduction

Exceptional longevity is influenced by a combination of environmental and genetic factors, and previous twin studies report that the heritability of human longevity is approximately 25% (Herskind et al., 1996). Family studies have suggested that exceptional aging tends to run in families, yet the search for genetic determinants of longevity has produced inconsistent results (Sebastiani et al., 2012; Sebastiani et al., 2013; Pilling et al., 2017). Several genome-wide association (GWA) studies have attempted to pinpoint genetic influences of healthy aging or longevity, yet only two loci, *TOMM40/APOE/APOC* and *FOXO3A*, have repeatedly reached genome-wide significance (Deelen et al., 2014; Broer et al., 2015). Thus, an alternative approach to understanding genetic factors underlying a complex phenotype like exceptional aging is warranted.

In our analysis, we utilized a comprehensive targeted sequencing approach designed to interrogate rare and common variants in both coding and non-coding regions within 20 key genes that are strongly associated with involvement in aging related processes and have utilized traditional statistical and machine learning approaches to explore aging-related genetic variants. The 20 genes were chosen because they have previously been associated with various molecular functions involved in aging, such as DNA damage response and repair, telomere maintenance, metabolism, cellular senescence, and stress resistance. There is ample evidence suggesting a causal role of DNA damage in aging and age-related diseases; for example most progeroid syndromes, including Werner syndrome, Cockayne syndrome (CS), and Fanconi anemia, are characterized by accelerated aging, possibly as a result of hypersensitivity to genotoxins predominantly due to problems with DNA repair and genome maintenance (Gensler and Bernstein, 1981; Hoeijmakers, 2009; Behrens et al., 2014; Vermeij et al., 2016).

Several lines of evidence also suggest that levels of DNA damage increase with age, whereas DNA repair capacity in mammals reduces with age (Niedernhofer et al., 2018). Comparative studies in mammals further indicate that species longevity positively correlates with DNA repair efficiency (Hart and Setlow, 1974; Tian et al., 2017; Ma and Gladyshev, 2017). Long-lived species such as the naked mole rat, *Heterocephalus glaber*, and bowhead whale, *Balaena mysticetus*, have a higher copy number of genes associated with DNA repair, possibly allowing for decreased susceptibility to age-accumulated DNA damage (Macrae et al., 2015; Tian et al., 2019). Therefore, we hypothesized that variants associated with DNA repair, telomere maintenance, and genomic stability could be predictive of phenotypic age.

Single variant association tests, such as linear regression, have been the statistical tools of choice for large GWA studies. In fact, many longevity-targeted GWA studies have taken this approach (Newman et al., 2010; Broer et al., 2015). However, such univariate models leave out epistatic effects that may be predictive of heterogeneous diseases, such as aging, resulting in frequent preclusion of the actual number of genetic factors contributing to or predictive of polygenic diseases (Stephan et al., 2015). More complex statistical approaches would account for genetic factors that alone have little association, but when considered in a multiplex manner, hold great predictive power. Random forest and support vector machines (SVMs) are just two of a multitude of ensemble learning methods capable of analyzing large data sets such as those obtained in GWA studies. Random forest couples bootstrap sampling and conditional inference trees for determining the importance of variables for classifying data (Lunetta et al., 2004). We sought to use random forest in our analysis of phenotypic aging, as it is capable of handling sizable data sets, considers the interactions between variables, and provides importance measures for predictors. On the other hand, SVM is a type of supervised learning that not only supports high dimensional data, but is robust against noise and sparsity in the data (Furey et al., 2000). SVM functions by taking a set of input features or data and defining an optimal decision boundary or hyperplane that most accurately separates the input space based on assigned binary classifiers. These factors allow for better determination of genetic predictors in polygenic diseases that might be due to nonlinear interactions in both common and rare variants, and was thus also implemented in our analysis (Lunetta et al., 2004).

For this study, we sequenced a panel of 20 aging-related genes with a targeted sequencing method previously developed by our lab in a cohort of 200 individuals selected from the University of Pittsburgh Claude D. Pepper Older Americans Independence Center (Day et al., 2014). Half of the cohort was labeled as phenotypic “early” agers, as determined by age (65–75 years old) and the inability to either walk up a flight of stairs or walk for 15 min without resting. The other half of the cohort was labeled as phenotypic “late” agers defined by age (>75 years old) and the ability to pass the walking tests performed on the early agers. We point out that regardless of grouping, all patients were ambulatory. In addition to gait, we also assessed multiple parameters of function, mental status, strength, and activity and number of diseases (comorbidity index).

After applying univariate and multivariate analyses to the sequencing data, we show that a decision tree-based method, random forest, trained on genetic markers in the discovery cohort shows promise in predicting phenotypic age. Despite the fact that a sample set of 200 is small for this genomic study, we show that our exploratory analysis to determine genetic predictors of aging provides a useful and novel mechanistic approach for investigating the association of polygenic risk variants with complex diseases. Further analyses with larger cohorts would find this approach valuable for determining a set of genetic variants, which when considered alone do not hold predictive value, but in combination are highly predictive of phenotypic aging. A predictive model of early phenotypic aging would not only give insight into key biological processes of this complex phenotype, but could potentially be used in a clinical setting as a diagnostic tool to indicate patients who may be at risk for early onset of age-related diseases.

## Materials and Methods

### Cohort Characteristics

#### Discovery Set UPMC (University of Pittsburgh Medical Center Cohort) Participants

Participants were recruited through the University of Pittsburgh Claude D. Pepper Older Americans Independence Center, which maintains a registry of more than 2,500 older adults who live in the greater Pittsburgh area and are interested in participating in clinical research. Print and radio ads were also used to recruit additional patients. Respondents were screened with a standardized phone interview. All study participants were community-dwelling and medically-stable volunteers who were independently mobile. Most respondents (~90%) were of self-reported Caucasian ethnic background (**Figures S1** and **S2**).

#### Assessments

- **Demographic information:** Age, gender, level of education, and smoking status.
- **Body composition:** Height, weight, and dual x-ray absorptiometry (DXA) to measure total fat and lean body mass.
- **Cognitive function:** Montreal Cognitive Assessment (MOCA) and Digit Symbol Substitution Test (DSST). Higher scores indicate better cognitive function.
- **General health:** Comorbidities were assessed using a comorbidity index (Rigler et al., 2002); a higher score suggests a greater number of comorbidities and poorer health (Sangha et al., 2003). The SF-36 measured patients' self-reported health and wellness; higher scores indicate better health (Ware and Sherbourne, 1992). Finally, participants were characterized as frail, prefrail, or robust using the five-item Fried Frailty Index; higher scores indicate frailty (**Figure S3**) (Abellan van Kan et al., 2008).
- **Function and activity:** We used the Community Healthy Activities Model Program for Seniors (CHAMPS) Physical Activity Questionnaire to assess the frequency of activity and estimate calories per week involved in the activity (Stewart et al., 2001). We assessed grip strength with a standard dynamometer. The short physical performance battery (SPPB) was used, which provides an integrated physical assessment based on several measures, including gait speed, chair stand, and balance; a higher score indicates better performance (Vasunilashorn et al., 2009).

#### Validation Set (Wellderly Cohort)

The Wellderly Cohort consists of individuals of at least 80 years of age with no chronic disease or need for chronic medications. Sample collection and processing for whole genome sequencing (WGS) as well as variant calling are as previously described (Erikson et al., 2016). Individuals used in this study had an average age of 86 and consisted of less males (n = 195) than females (n = 316). Comparison of overlapping clinical features in the discovery and validation cohorts were assessed to ensure a similar population distribution (**Figures S4A, B**). Furthermore, the cohort contains no enrichment for longevity variants.

#### Participant Group Determination

We sought to maximize the signal with respect to any genetic differences between the groups. Because there is no standard operational criterion for defining early and late agers, we used self-reported and performance-based measures of mobility (Abellan van Kan et al., 2008), strongly associated with incident functional decline, disability, and mortality in the elderly (Perera et al., 2016). As such, we operationally defined “early aged” participants as those 65–75 years of age who could not walk up a flight of stairs or walk for 15 min without resting; “late aged” were those age 75 years and older who could walk up a flight of stairs or walk for 15 min without resting. The age cut-off of 75 years was chosen as it has been utilized in numerous phenotypical aging studies in older adults (Boonen et al., 2006; Boonen et al., 2010; McClung et al., 2012). We excluded participants with a history of a major cancer. **Table 1** depicts the differences in participant characteristics between groups.

**Table 1:** Comparisons of variables between aging cohorts: mean ± standard deviation.

Demographics	Early aged 65–75 (n = 100)	Late aged >75 (n = 100)	Early *vs.* late aged p-value*
Age (years)	70.4 ± 3.0	83.2 ± 5.4	<0.0001
Sex (% female)	63 (63.0)	56 (56.0)	0.3133
Comorbidity index (of 14 conditions)∆	4.4 ± 1.8	2.5 ± 1.6	<0.0001
Arthritis	68%	45%	<0.001
Gait Speed (m/s) #	0.92 ± 0.24	1.08 ± 0.26	<0.0001
% used cane	14%	5%	<0.001
% used other device	5%	1%	
BMI (mean; kg/m2)	33.5 ± 8.3	27.2 ± 4.6	<0.0001
BMI ≥ 40	24%	1%	<0.001
Lean body mass (kg)	53. ± 11.8	47.4 ± 9.9	0.0002
Total mass (kg)	91.6 ± 23.5	73.6 ± 15.3	<0.0001
% Fat body mass	37.9 ± 8.6	32.2 ± 7.8	<0.0001
MOCA (1–30) #	25.3 ± 2.8	24.3 ± 3.5	0.03
DSST Score #	42.2 ± 9.5	39.7 ± 10.7	0.0808
Grip strength (kg) (dominant)	26.7 ± 10.8	26.7 ± 10.6	0.9791
Chair rise time (s)∆	14.7 ± 13.8	12.4 ± 11.8	0.0001
SPPB total score #	9.1± 2.5	10.2 ± 1.8	0.0005
Balance score #	3.4 ± 1.0	3.6 ± 0.7	0.1873
Calories from all activity per week	2320 ± 2186	3585 ± 3059	0.001
Calories from moderate activity per week	929 ± 1495	2018 ± 2322	0.0001
Freq of all activity per week	13.9 ± 9.8	19.7 ± 10.6	<0.0001
Freq of moderate activity per week	4.3 ± 5.0	7.2 ± 6.4	0.0003
Frail scale ∆	2.6 ± 1.3	0.6 ± 0.9	<0.0001
Physical function index #	37.3 ± 19.1	77.2 ± 17.2	<0.0001
General health perception #	52.8 ± 22.2	78.2 ± 14.3	<0.0001
Bodily pain #	44.5 ± 22.4	76.3 ± 19.7	<0.0001
Social function #	69.0 ± 24.5	92.1 ± 15.7	<0.0001
Mental health index #	65.2 ± 14.3	75.8 ± 9.4	< 0.0001
Vitality #	47.7 ± 14.1	66.5 ± 11.8	<0.0001

*Computed using independent samples t-, Wilcoxon rank sum, or chi-square tests, as appropriate. ∆ Lower score better, # Higher score better.

### Variant Genotyping

Clone adapted template capture hybridization sequencing (CATCH-Seq) was used as an alternative to other sequencing methods due to the low cost and high coverage ability of both coding and noncoding genomic regions (Day et al., 2014). CATCH-Seq yield is comparable to WGS (89 *versus* 98% at 100x) but at a fraction of the cost, allowing for more samples to be included in a study when only a small set of genes are under investigation, as is the case in this investigation. CATCH-Seq probes were designed to capture ~150–200 kilobase (kb) regions around each of the 20 target genes (**Table 2**). Standard Illumina sequencing libraries were hybridized to the CATCH-Seq probes, and the target-enriched libraries were subjected to 2 x 100 base pair (bp) paired-end sequencing on HiSeq 2500 sequencers. The resulting sequence data was aligned to the human reference genome (GRCh37) with Burrows-Wheeler Aligner (BWA) (Li and Durbin, 2009), and variants were called using GATK v2 (McKenna et al., 2010) with exclusion filters for variants with low mapping quality (mapq < 20) and low genotype quality (q < 30).

**Table 2:** Names, biological function, and literature references for aging association of the 20 genes sequenced.

Gene	Function	Biological association with aging/age-related pathology	Literature reference for study inclusion
*Apolipoprotein E* (APOE)	Combines with lipids to form lipoproteins, which package cholesterol and other fats for transfer through the bloodstream.	Polymorphisms in APOE are associated with human longevity.	(Broer et al., 2015; Soto et al., 2015; Pilling et al., 2016)
*Aprataxin* (APTX)	Involved in DNA break repair and base excision repair.	Defects in aprataxin cause the autosomal recessive neurodegenerative disorder ataxia oculomotor apraxia 1 (AOA1).	(Katyal and McKinnon, 2008; Krishnan et al., 2011; Coppede and Migliore, 2012)
*Bloom syndrome RecQ like helicase* (BLM)	ATP-dependent DNA helicase. Unwinds DNA in the 3'-5' direction. Involved in double-strand break repair.	Defects associated with segmental aging of the immune system together with an elevated risk of otitis media and pneumonia, an elevated risk of diabetes mellitus, reduced fertility, and higher cancer incidence.	(Karow et al., 2000; Coppede and Migliore, 2012; de Renty and Ellis, 2016)
*Cyclin dependent kinase inhibitor 2A* (CDKN2A)	Induces cell cycle arrest and acts as a tumor suppressor.	Mutations near CDKN2A were particularly associated with diseases of aging (e.g., cancer, atherosclerosis, type 2 diabetes, glaucoma). CDKN2A expression increases with age. Removal of p16+ cells in mouse models increases health span and lifespan.	(Baker et al., 2008; Shiels, 2010)
*Sialic acid binding Ig-like lectin 3* (CD33)	Mediates cell-cell interactions and maintenance of immune cells in the resting state.	Mutations in CD33 are associated with AD risk.	(Griciuc et al., 2013; Estus et al., 2019)
*Dyskerin pseudouridine synthase 1* (DKC1)	Stabilization and maintenance of telomerase.	Mutations in DKC1 causes premature aging, bone marrow failure, and cancer.	(Blasco, 2007; Gu et al., 2011)
*Excision repair cross-complementing rodent repair deficiency, complementation group 4* (ERCC4)	Catalytic component of a DNA repair endonuclease responsible for 5' incision during DNA repair.	Loss of ERCC4 causes systemic accelerated aging (XPE) and neurodegeneration.	(Muñoz et al., 2005; Bogliolo et al., 2013; Yuan et al., 2014)
*Excision repair cross-complementing rodent repair deficiency, complementation group 5* (ERCC5)	Endonuclease involved in single-strand DNA nucleotide excision repair at the 3' end.	Mutations in ERCC5 lead to Cockayne Syndrome (CS), which is characterized by premature aging.	(Coppede and Migliore, 2012)
*Excision repair cross-complementing rodent repair deficiency, complementation group 6* (ERCC6)	DNA-binding protein involved in transcription-coupled nucleotide excision repair.	Defects in ERCC6 cause CS and age-related macular degeneration.	(Tuo et al., 2006; Baas et al., 2010)
*Fanconi anemia group A protein* (FANCA)	DNA repair protein involved in Interstrand Crosslink (ICL) repair.	Defects cause Fanconi anemia, a progeroid syndrome with symptoms common in premature aging (sarcopenia, hypersensitivity to infectious agents, endocrine abnormalities, etc.).	(Soria-Valles and López-Otín, 2016; Schumacher et al., 2008)
*Lamin A/C* (LMNA)	Component of the nuclear lamina.	LMNA mutations cause Hutchinson-Gilford syndrome (HGPS).	(Rodriguez et al., 2009; Kawahara et al., 2011; Lopez-Mejia et al., 2011)
*Poly(ADP-ribose) polymerase 1* (PARP1)	Mediates poly-ADP-ribosylation of proteins and plays a role in DNA repair, chromatin remodeling, telomere maintenance, and mediator of inflammation.	PARP1 activation increases with age in *C. elegans*. Increased activation has been associated with aging, neurodegeneration and metabolic abnormalities in humans.	(Krishnan et al., 2011; Coppede and Migliore, 2012; Maynard et al., 2015)
*DNA polymerase beta* (POLB)	DNA polymerase involved in base excision and repair.	*Polb^+/−^* mice have an increased age-related mortality rate and tumorigenesis.	(Strosznajder et al., 2000; Cabelof et al., 2002)
*DNA polymerase gamma* (POLG)	Involved in mitochondrial DNA replication.	Increased mitochondrial mutation load in mice is associated with premature aging.	(Trifunovic et al., 2004; Kujoth et al., 2005; Hiona and Leeuwenburgh, 2008)
*Sirtuin 1* (SIRT1)	NAD-dependent protein deacetylase. Involved in cell cycle regulation, response to DNA damage, metabolism, apoptosis, and autophagy.	SIRT1 overexpression extends lifespan in mice. Mutations are associated with age-related pathologies such as myocardial infarction (MI).	(Grabowska et al., 2017; Satoh et al., 2013; Yuan et al., 2016)
*Sirtuin 6* (SIRT6)	NAD-dependent protein deacetylase. Deacetylase activity toward histones H3K9Ac and H3K56Ac. Required for genomic stability. Deacetylates telomeric DNA.	SIRT6 overexpression extends lifespan. Long-lived animals have highly efficient SIRT6 function.	(Schumacher et al., 2008; Berman et al., 2012; Serrano et al., 2013; Moskalev et al., 2014)
*Superoxide dismutase 2* (SOD2)	Destroys superoxide anion radicals produced in cells.	SOD2 mutations are associated with heart disease and increased risk of malignancies.	(Patel, 2002; Fabrizio et al., 2004; Qiu et al., 2010; Velarde et al., 2012)
*Telomerase reverse transcriptase* (TERT)	Ribonucleoprotein polymerase that maintains telomere ends by the addition of the telomere repeat TTAGGG.	Telomere attrition is highly associated with aging due to increased cellular senescence.	(Aubert and Lansdorp, 2008; Martinez and Blasco, 2010; Ghosh and Zhou, 2014; Blackburn et al., 2015)
*TERF1 interacting nuclear factor 2* (TINF2)	Component of the telosome that is involved in telomere length regulation and protection.	Mutations in TINF2 are linked to Revesz syndrome, a telomeropathy with symptoms characteristic of accelerated aging.	(Kim et al., 1999; Rubelj and Vondraček, 1999; Savage et al., 2008)
*Werner syndrome RecQ like helicase* (WRN)	DNA helicase that is involved in maintenance of genomic stability, DNA repair, replication, transcription, and telomere maintenance.	Mutations in WRN lead to Werner syndrome with systemic aging phenotypes.	(Mohaghegh and Hickson, 2002; Ding et al., 2007; Multani and Chang, 2007; Bendtsen et al., 2012)

### Quality Control

#### Variant Inclusion Criteria

The initial datasets consisted of 25,273 variants in the discovery cohort and 8,018 variants in the validation cohort (**Table S1**). Rare variants, or those with less than eight alleles observed in the discovery cohort, and those with variants with over 10% missing data, were excluded. Variants not covered in both the discovery and validation cohort were also excluded. Variants were then imputed across individual genes +/− 50 kb using K-nearest neighbor imputation *via* the impute package in R (Hastie et al., 2001). A total of 5,896 variants was selected for further analysis.

### Statistics

#### Total Variance Analysis

The sum of all variance between groups was analyzed using a Wilcoxon rank-sum test to determine whether “early” agers had more or less genetic variance in the target genes compared to “late” agers.

#### Single Variant Association

Logistic regression was utilized to assess the association of any single variant to the age group phenotype. A quantile-quantile (QQ) plot was used for evaluation of the distribution of p-values.

#### Gene Association

Wilcoxon rank-sum tests were used to compare the distribution of Combined Annotation-Dependent Depletion (CADD) scores of non-reference alleles near target genes (+/− 50 kb) between early and late agers. P-values were adjusted for multiple hypothesis *via* the Bonferroni method.

#### Predictive Modeling

Four-fold cross-validation with four different seeds using a random forest regression model *via* the RandomForest package in R as well as SVM classification *via* the e1071 package in R were conducted for predictive modeling of the aging phenotype (Liaw and Wiener, 2002; Dimitriadou et al., 2005). Default settings for number of trees grown (n = 500) and number of variables tried at each split (mtry = 6) were used for each random forest model. An SVM model was tuned using a range of costs (c = 0.1, 1.0, 10.0, 100.0) and gamma values (gamma = 0.5, 1, 2). Both random forest and SVM modeling were performed on 28 different stratifications of the data in addition to a control data set (**Table S2**) resulting in 928 models in total. Most of the data subsets consisted of different groups of genomic spaces within the sequenced data as well as filters for frequency and deleteriousness. The first subsets of the data contained all sequence variants in addition to groups with different filters, including a subset of rare variants (tAF < 0.1), very rare variants (tAF < 0.01), mildly deleterious, and highly deleterious variants as defined by the CADD score (CADD > 10 and CADD > 15, respectively). We then took subsets of only the variants within the start and end site of the target genes and then applied the same filters as the first to analyze rare (tAF < 0.1), very rare (tAF < 0.01), mildly (CADD > 10), and highly (CADD > 15) deleterious variants. The next set of subsections contained target gene variants plus 50 kb up- and down-stream of the transcription start and end sites to capture regulatory genomic space within the analysis. Once again, the same cutoffs for allele frequency and CADD score were applied. The last genomic space stratification included variants within exons of the target gene isoforms, thus eliminating intronic space from the models. Allele frequency and CADD score cutoffs further stratified the exonic variant subset. In addition to stratifications of the genomic space, publicly available databases such as the Genome-Wide Repository of Associations Between Phenotypes (GRASP), the single nucleotide polymorphisms (SNP) and copy number annotation (SCAN) database, and software such as SIFT (sorting intolerant from tolerant) were utilized for grouping the data based on variant effect (Ng and Henikoff, 2003; Gamazon et al., 2010; Lonsdale et al., 2013; Leslie et al., 2014). For this, we analyzed known versus unknown variant models, SIFT deleterious variants *vs.* SIFT tolerated variants, variants effecting expression, and GWAS variants. We also included a control set, which was made by randomly shuffling all of the variants. We did not adjust for age in any of the models tested, because age should not affect this analysis of SNPs.

We assessed the performance of each model using receiver-operating characteristics (ROCs). Additionally, we used Bayesian Classifier to determine the optimal cut-off between early and late agers in the random forest regression analysis. Top performing SVM and random forest models were tested on the validation (Wellderly) cohort of late agers, and the misclassification percentage, based on the optimal cut-off, was used to rank each model rather than ROC-area under the curve (AUC), since the cohort comprises a single class (late agers) rather than the binary class available in the discovery cohort. Top classifiers in the best performing random forest model were determined by analyzing the Gini importance measures (Gini coefficient) for each split in the top models, which gives a measure of variable importance. In other words, the higher the Gini coefficient, the better the classifier is at accurately splitting the data between two classes.

#### Enrichment Analysis

Enrichment for specific genomic domains and functions within the top variants was determined using a variety of tools. Enrichment of rare or severely deleterious variants was analyzed by assessing allele frequency and CADD scores of the top variants. We utilized the UCSC Genome Browser for determination of the specific location of each variant for analysis of intronic or exonic SNP enrichment (Kent et al., 2002). GRASP was used to discover whether the top classifying SNPs have been previously associated with specific phenotypes (Leslie et al., 2014). The Roadmap Epigenomics Project database was used to ascertain how many top variants were within regulatory regions *via* data from the HepG2 hepatocellular carcinoma cell line as well as GM12878 lymphoblastoid cells (Chadwick, 2012). Lastly, enrichment for transcription factor binding sites within the top 50 variants was assessed using data from the ENCODE database (ENCODE Project Consortium, 2012).

## Results

### Clinical Characteristics

As expected by design, and despite its older age, the late aging group had better scores for gait speed, chair rise time, SPPB, physical function, self-perceived health, bodily pain, social function, mental health, and vitality (all p < 0.05). The late aging group was also less likely to suffer from comorbidity or frailty (p < 0.05), expended more calories from all activity per week and from moderate activity per week, and displayed a higher frequency of all and moderate activity. However, cognitive scores were similar between the two groups.

### Logistic Regression

**Figure 1:**
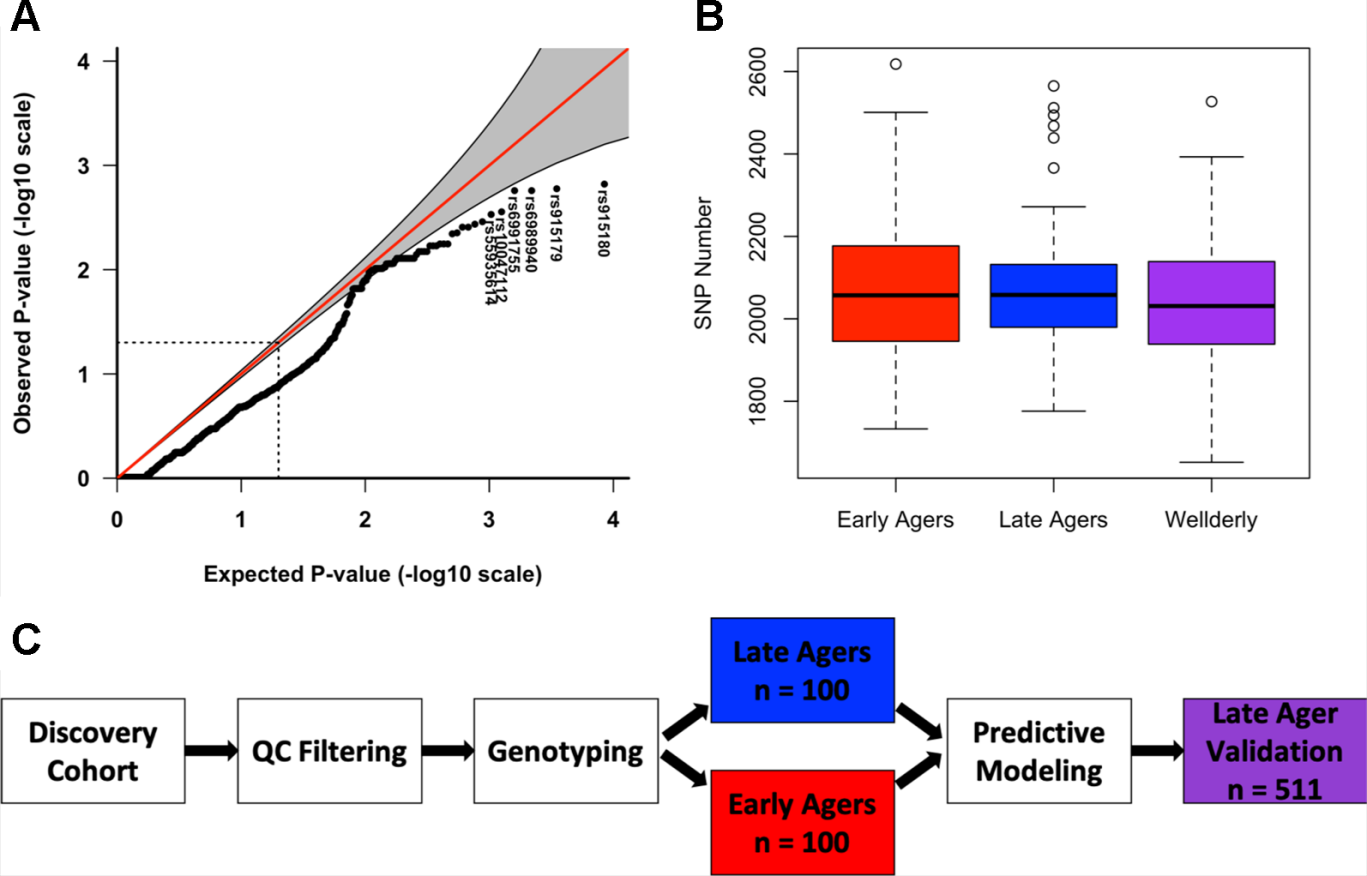
Logistic regression and variant burden reveal lack of association with early aging. **(A)** Quantile-quantile plot of logistic regression p-values. **(B)** Box plot of total number of variants in the discovery early aged group (red), discovery late ager group (blue), and the validation late ager group (purple). **(C)** Diagram of predictive modeling analysis study design.

To identify high-impact aging-related variants, variants were tested for association with the aging group using logistic regression. Top variants were within intronic and upstream regions of *lamin A* (*LMNA)* (rs915180, p value = 0.0015) and *Werner syndrome ReqQ like helicase* (*WRN)* (rs6989940, p value = 0.0017), however none of the top hits reached significance beyond what would be expected by chance given the number of individual variant tests. A QQ plot of the logistic regression p-values indicated deflation as a result of a lack of power owing to the small sample size in this study (**Figure 1A** and **Table S3**).

### Variant Burden

We combined the number of alternate alleles among all 20 genes in each subject following simple inclusion criteria of the variants for quality control to determine if early agers had a larger variant burden in aging-related genes compared to late agers, finding no significant difference (Wilcoxon p value = 0.75) (**Figure 1B**). This method was then repeated for each individual gene, for which we compared the total amount of non-reference alleles in early agers compared to late agers in order to test whether the variant burden in that gene differed between groups. There was little difference in total non-reference allele count per target gene between early and late agers for most of the genes analyzed (**Figures S5A**, **B**). However, *LMNA* approached the Bonferroni corrected p-value of 0.003 according to a Wilcoxon rank-sum test (p-value = 0.006, FDR = 0.1).

### Machine Learning

Since neither the univariate nor the gene-based multivariate analyses yielded statistically significant associations with aging group, we moved on to a computational approach geared at determining the predictive power of our sequencing data for aging status. Both random forest and SVM were applied to the variant data to determine the best genetic predictors of late aging. The ability of both the random forest algorithm and SVM to outperform other non-parametric classification methods led to our use of these predictive modeling approaches in this study (Furey et al., 2000; Lunetta et al., 2004). As depicted in **Figure 1C**, the training cohorts were divided into early and late agers for random forest model training, and top performing models according to the ROC-AUC were then tested for prediction of aging status in the validation cohort. Various stratifications of the data were fed into each algorithm to determine the best subset of predictors. These subsets included: variants of both low and high allele frequencies, variants that are known to effect expression (eQTL) defined by the SCAN database, variants previously associated with aging as determined by the GRASP database, functional variants determined by ENCODE, variants with low and high levels of deleteriousness as defined by the CADD scores, and variants near or within the target genes. Four-fold cross-validated random forest at four different seeds was performed on these various filters of the variant data as previously described, resulting in a total of 16 models per filter, or 464 total models.

**Figure 2:**
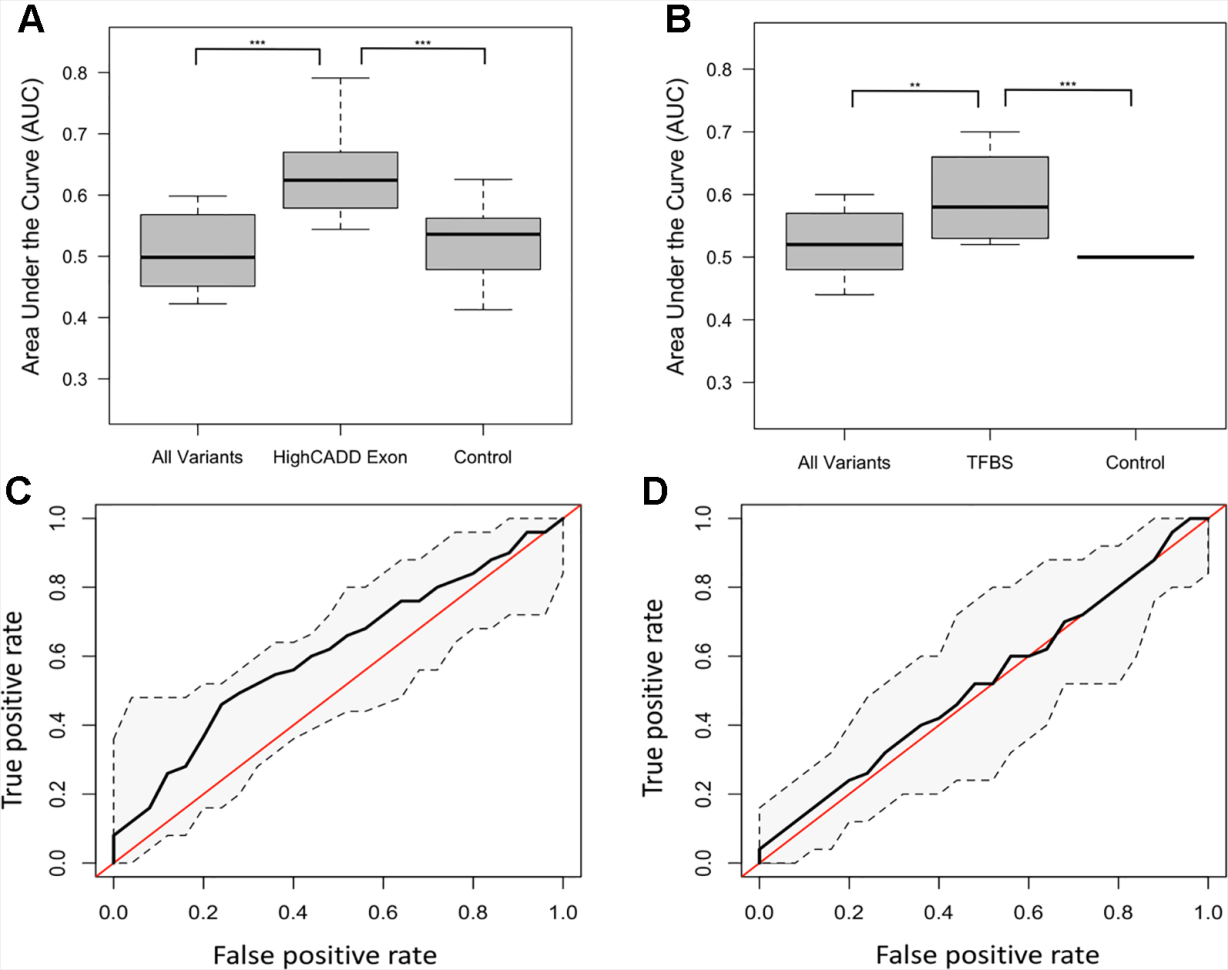
Different subsets of variants defined as top predictive models using random forest and support vector machine (SVM) learning methods. **(A)** Boxplots of the random forest model area under the curve (AUCs) for the all variant, high Combined Annotation-Dependent Depletion (CADD) exon and control subsets of the variant data. P-values between groups determined by performing a Kruskal-Wallis test. **** = p < 0.0001, *** = p < 0.001. **(B)** Boxplots of the SVM model AUCs for the all variant, transcription factor binding site (TFBS), and control subsets of the variant data. P-values between groups determined by performing a Kruskal-Wallis test. **(C)** Receiver-operating characteristic (ROC) curve of the mean high CADD exon random forest model with confidence intervals. The red line represents the null AUC (0.5). **(D)** ROC curve of the mean TFBS SVM model with confidence intervals. The red line represents the null AUC (0.5).

**Figure 3:**
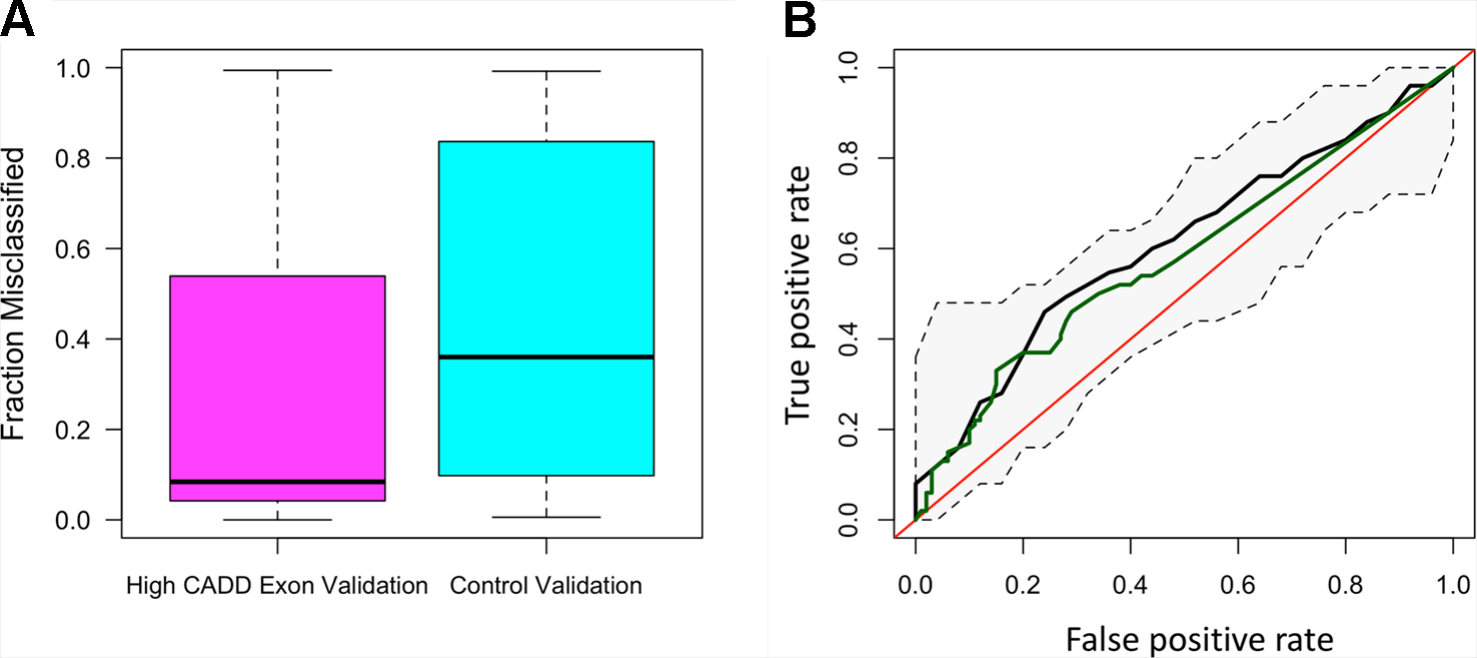
The random forest high Combined Annotation-Dependent Depletion (CADD) exon model is predictive of late aging status in the validation cohort and outperforms smoking as a predictor of aging. **(A)** Boxplots of the fraction of misclassified patient samples based on the random forest high CADD exon model (magenta) and the control random forest model (shuffled dataset) (teal). **(B)** Receiver-operating characteristic curve of the mean area under the curve (AUC) resulting from the random forest high CADD exon model (black) with confidence intervals in the discovery cohort and the AUC resulting from smoking status as a sole predictor of early *versus* late aging (green).

The distribution of ROC-AUCs, a measure of model sensitivity and specificity, was compared to identify the top performing models (**Table S4**, **Figure S9**). Random forest performed on the non-reference alleles within the exons of the 20 target genes having a CADD score greater than 15 showed the greatest performance (mean ROC-AUC = 0.62) among random forest models, while the model trained on non-reference alleles within TFBSs proved to have the highest performance among all SVM models (**Figures 2A**–**D**), but failed to outperform the top random forest model. Furthermore, the top random forest model outperformed the random forest model trained on the control shuffled data set (Wilcoxon p-value = 1.5x10^−4^), demonstrating that despite having a mean AUC of 0.62, the model performs significantly better than the control model. This model also proved to outperform that of all sequenced variants (mean ROC-AUC = 0.51) (Wilcoxon p-value = 9.5x10^−5^). For analysis of model predictive power in an independent cohort, we tested the ability of the top random forest model to correctly identify the validation (Wellderly) cohort as late agers. As previously stated, because this cohort lacked any early agers, we used percent misclassification rather than ROC-AUC to assess prediction accuracy as ROC-AUC assessment requires two groups. This analysis revealed that the top model performed well on the model validation (Wellderly) cohort (median misclassification = 0.02) (**Figure 3A**). Additionally, smoking status, which is known to affect aging, was tested as a predictor of age group for comparison of genomic data to environment in predicting aging status, revealing that our model built on high CADD exon variants in aging-related genes performed comparably (**Figure 3B**) (Valdes et al., 2005; Csiszar et al., 2009; Astuti et al., 2017). Because there is a significant difference in BMI between early and late agers (p = 5.6 x 10^−8^), we tested the correlation between the predictor value and BMI in the discovery cohort for the top performing model, which revealed little correlation between age group prediction and BMI (Spearman Rho = 0.07) (**Figure S6**). Furthermore, a scatterplot of the predicted age group from the best model (mean ROC-AUC = 0.62) versus BMI in both cohorts details a lack of trend between the two values, further supporting that this is a model predictive of early versus late aging rather than BMI (**Figure S7**).

One of the most advantageous aspects of the random forest, especially when predicting phenotypes, is that it returns importance scores for each predictor in the model, allowing for the ranking of classifiers within the dataset and associations between predictors and phenotypes. Classifiers in the top performing model were ordered by their Gini coefficient, a measure of how well the classifier contributed to accurately separating the classes. We found that the predictors within the top performing model (high CADD exon variants) were nonsynonymous mutations within 9 of the 20 genes (*APTX, BLM, ERCC4, ERCC5, ERCC6, LMNA, PARP1, POLG*, and *WRN*).

### Enrichment Analysis

**Figure 4:**
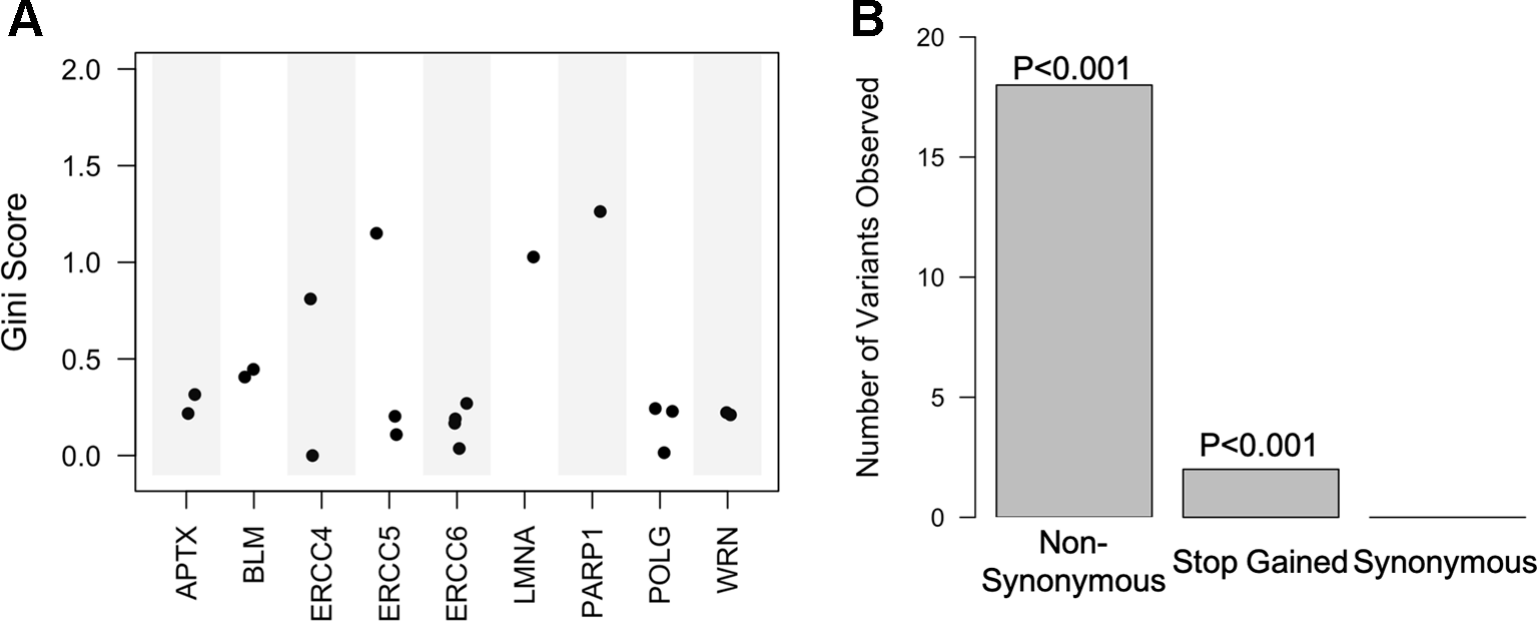
Random forest high Combined Annotation-Dependent Depletion exon predictive variants are within 9 of the 20 genes and mostly non-synonymous. **(A)** Scatter plot of the Gini score for each of the predictive variants based on corresponding gene. **(B)** Bar plot of the variant consequence type within the predictors with corresponding empirical p-values.

Top variants were determined by averaging the Gini coefficients across the 16 models performed on the highly deleterious target gene exon data set. Enrichment analysis was then conducted on these variants in regard to gene and variant effect. We found that a majority of the top variants were located within *excision repair cross complementation group 4* (*ERCC4*)*, ERCC5, LMNA*, and *PARP1* (**Figure 4A** and **Table S5**). Furthermore, 6 of the predictor's regions have previously been associated with more than 15 different phenotypes in the GRASP database (**Table S6**). Enrichment analysis of variant consequence effect revealed that predictors are enriched for those that cause a nonsynonymous change as well as a stop gain, or premature termination codon (p < 0.001) and depleted for synonymous mutations (**Figure 4B**).

## Discussion

Although aging is highly dependent on environmental, behavioral, and social interactions, studies have shown that a quarter of the variance explaining longevity is heritable (Herskind et al., 1996; Perls et al., 2002; van den Berg et al., 2018; van den Berg et al., 2019). Yet, only a handful of genetic determinants explaining a small portion of the heritability have been discovered to date. Hampering additional discovery are the complexity of the biology of aging as well as the rarity of the longevity phenotype. Analysis of late aging rather than longevity allows for larger cohort sizes, as late agers are more common in the general population than long-lived individuals (>100 years old); however, the lack of a clear definition for “healthy” or late aging makes genetic analysis and cross-study interpretation of this phenotype extremely difficult. Recently, Reed et al., defined “healthy” aging as living to the age of 70 in the absence of coronary surgery, heart attack, stroke, diabetes, or prostate cancer, finding an approximate 50% heritability of the defined phenotype in a cohort of male twins (Reed et al., 2004). Several other late aging cohorts exist, characterized by various definitions and resulting in inconsistent heritability percentages and gene associations (Walter et al., 2011; Brooks-Wilson, 2013; Erikson et al., 2016). Furthermore, large-scale aging GWA studies to date have failed to identify recurrent specific genomic regions that statistically associate with the longevity or late aging phenotypes, though combined analysis of SNPs have identified pathways and multi-allele signatures associated with aging phenotypes, indicating that these studies should include polygenic or epistatic associations in addition to the more traditional analysis of single gene associations to more successfully discover genetic determinants of aging phenotypes (Brooks-Wilson 2013). This observation led us to design a unique approach for determining genetic predictors of phenotypic aging by conducting targeted sequencing of 20 previously determined aging-related genes in a cohort of “early” and “late” agers. This approach allowed for the identification of a set of genetic variants associated with various aspects of genomic integrity as possible predictors of late aging. While we emphasize that our small discovery cohort (n = 200) is not ideal for a genomic association study, our process of combining targeted sequencing and machine learning to identify a set of genetic factors that together act as predictive determinants for a complex disease will be useful in further genetic association studies of complex phenotypes for which individual variant association is insufficient.

Our initial analysis of overall variant burden and individual variant association with early versus late aging failed to produce any variant with statistically significant association. While this is typical of GWA studies, especially those with a complex phenotype or small sample sizes, single variant association does prove useful for prioritizing variants by p-value. In our analysis, two intronic variants within *LMNA* had the strongest association (rs915180 and rs915179) and were also the most predictive variants in the unfiltered data set random forest models (**Figure S8**). These variants have also been previously associated with longevity (**Table S3**). In fact, rs915179 is part of a haplotype within *LMNA* that is specifically associated with longevity (Conneely et al., 2012). Sebastiani *et al*., used rs915179 as part of a “genetic signature” of exceptional longevity and later confirmed this variant in a meta-analysis of longevity (p = 0.0001) (Sebastiani et al., 2012). *LMNA* encodes lamins A and C, which are nuclear envelope proteins. These proteins are associated with Hutchinson-Gilford progeria syndrome (HGPS), an extremely rare disease causing premature aging and with a life expectancy of about 13 years (Conneely et al., 2012). Interestingly, defective forms of *LMNA* are produced in small amounts within cells of healthy individuals, and there is evidence that this amount increases with age (Rodriguez et al., 2009). This variant was also one of the first to be associated with Alzheimer's disease in GWA studies, indicating that it may play a pivotal role in cognitive function, which is known to decline with increasing age. In GWA studies, rs915180 has been associated with suicide attempts in patients with mood disorders, as well as with cardiomyopathy, chronic kidney disease, and birth weight (Perlis et al., 2010; Köttgen et al., 2010; Horikoshi et al., 2013). Since this association failed to reach genome-wide significance, future studies involving larger cohorts are needed to further assess the association of rs915179 with late aging.

Because the individual variant association proved inadequate for determining variants within our data that are predictive of aging status, we next focused our analysis on machine learning. Random forest and SVM were performed on various stratifications of the data, and assessment of the resulting ROC-AUC and misclassification percentages revealed that the random forest model built using variants with a CADD score over 15 (high CADD) proved to be the best performing predictor of aging status. As previously noted, one of the benefits of using random forest is that it ranks predictors based on how well they add to the purity of the model (Gini coefficient). The mean coefficient for each predictor in all trials of the high CADD exon variants was used as a metric with which to rank variants (**Figure 4A**). The variant with the highest predictive power (rs1136410) in our top performing model of aging status is located in *PARP1* and causes an A > G alteration in the 17th exon (mean Gini = 1.26). *PARP1* is responsible for posttranslational modification of nuclear proteins in response to various types of DNA damage as well as oxidative stress (Muiras et al., 1998; Beneke and Bürkle, 2007). With an essential role in base excision repair (BER) and double strand break (DSB) repair, *PARP1* has been known as the “sensor of nicks” within DNA (Mao et al., 2011; Czarny et al., 2017). Comparative studies among 13 mammalian species found that the enzymatic activity of PARP1 positively correlates with maximum lifespan in various mammals, including humans (Bürkle et al., 1992; Muiras et al., 1998; Piskunova et al., 2008; Noren Hooten et al., 2012). Additionally, this variant has previously been associated with survival in patients with early stage non-small-cell lung cancer, depression, and baseline hippocampal volume loss in apolipoprotein E genotypeε4 (*APOE4*) (Nho et al., 2013).

The next strongest predictor in the top performing model is located within *ERCC5/XPG* (mean Gini = 1.15), located on chromosome 13q22–33 which causes a G > C (His1104Asp) change in the last (15th) exon of the gene (rs17655) (Zhao et al., 2018). *ERCC5* is an excision repair gene that is responsible for forming the 3' incision during nucleotide excision repair (NER) and is known to be extremely polymorphic (Zhao et al., 2018). The variant is located within the C-terminal of the gene and inhibits interactions of *ERCC5* with other DNA repair proteins (Xu et al., 2016). Damaging variants in this gene can lead to deficiencies in the NER pathway, causing xeroderma pigmentosum (XP) and Cockayne syndrome (CS), both of which result in symptoms shared with phenotypic aging (O'Donovan et al., 1994; Barnhoorn et al., 2014). Additionally, this specific variant, rs17655, is well-studied for its association with cancer risk, especially in gastric and colon cancer (Zhao et al., 2018). The well-established relationship between accelerated aging and deficient DNA damage repair (Gensler and Bernstein, 1981), in addition to the high importance this variant has in our top performing model, leads to the hypothesis that *ERCC5* is important for attenuating the aging process.

The next most important variant in the predictors is within *LMNA* (rs513043), which causes a missense mutation (G > A) in the 2^nd^ codon and has a CADD score of 18.44, indicating a high degree of deleteriousness (mean Gini = 1.03). *LMNA* encodes nuclear proteins lamins A and C for which mutations in this gene are associated with numerous diseases including cardiomyopathies, lipodystrophy, muscular dystrophies, and progeroid (early aging) syndromes, such as HGPS. Again, the nuclear lamina has been repeatedly linked to aging; in fact, Sebastiani et al., used numerous *LMNA* variants to build a “genetic signature” of longevity (Sebastiani et al., 2012).

Lastly, a variant in *ERCC4* (rs1800067) was also one of the top predictors in the best predictive model (mean Gini = 0.81). This variant causes a missense mutation (G > A) in the 8th exon, has a CADD score of 36, indicating a very high degree of deleteriousness within the gene, and has been associated with HDL cholesterol and risk of glioma and lung cancer. *ERCC4* is an excision repair gene that forms a heterodimer with *excision repair cross complementation group 1* (*ERCC1*) for NER. Reduced expression of *ERCC4-ERCC1* leads to XPF-ERCC1 (XPE) progeria in humans that is characterized by systemic accelerated aging (Niedernhofer et al., 2006). Moreover, other studies examining genes under positive selection in the longest-lived mammalian species, the bowhead whale, identified *ERCC1* as a top hit, suggesting that this pathway may promote maintenance of health (Keane et al., 2015). Jorgensen et al., showed that this variant is significantly associated with benign breast disease (BBD), especially in patients with a family history of breast cancer (Jorgensen et al., 2009).

Like many genomic studies of longevity and late aging, several limitations of this study warrant comment. First, in the absence of field-wide consensus regarding the definition of early versus late aging, we relied on physical function to differentiate the two groups. The parameters used to differentiate them—the ability to walk 15 min without stopping and to climb a flight of stairs—are well-validated (Abellan van Kan et al., 2008; Perera et al., 2014; Perera et al., 2016) and can be viewed as integrative, i.e., incorporating the impact of both physiological decline and diseases. The advantage of using such standardized assessments of function is the ability to differentiate participants into non-overlapping groups. The disadvantage is that impaired function may reflect the effect of not only early aging, but also comorbidity. However, because aging is characterized by both constriction of physiological reserve and the accumulation of diseases, it is difficult to disentangle the impact of early aging and disease. It is possible that subtle effects of genes or alleles on aging were masked by the impact of superimposed diseases, but testing this hypothesis will require a study large enough to identify a sufficient number of participants who qualify as early agers in the absence of disease. It is also possible that conditions such as comorbidity, obesity, and frailty lie in the causal pathway from any genetic predispositions to functional outcomes. Therefore, efforts to control for them would attenuate any associations between genetics and the function-based group definition. Another limitation of this study, which is common among many genomic studies, is the cross-sectional design; future studies are needed to examine longitudinal trajectories. Lastly, a validation cohort consisting of both early and late agers would improve our confidence in the constructiveness of this model for both early and late aging phenotypes.

## Conclusion

In conclusion, the two assessments regarding walking and stair climbing helped to identify a group of phenotypically late agers who had a better gait speed, higher activity and greater activity as well as physical function compared to a group of phenotypic early agers. This study found that more complex statistical analyses encompassing epistatic effects rather than traditional single gene association tests are useful for interpretation of rare genomic data generated using deep sequencing methods. Random forest provided information complementary to more traditional statistical analyses, including the ability to correctly classify the validation cohort of late agers 90% of the time. Predictors in the model were within genes that are involved in DNA repair and stability. We recognize that there are many genes and possibly intergenic regions of the genome engaged with genome stability and the biology of aging that were not included in this study; however, the genes chosen for analysis here are those with which the authors have had the greatest familiarity and sequence knowledge. Additionally, while we did not account for admixture in our analysis, we believe this would not drastically alter our results, as most of our discovery cohort and the entire validation cohort were of self-reported European American descent. While we realize that the training set has a low number of patients to achieve statistical certainty, we propose that holistic analysis of rare variant data may have promise in a larger cohort. Thus, targeted sequencing of genes involved in aging in combination with machine learning should be considered as a method to determine predictors of complex phenotypes.

## Data Availability Statement

The raw sequencing data in this manuscript is available at NCBI (SubmissionID: SUB6523418 BioProject ID: PRJNA589212).

## Ethics Statement

Patient samples were collected with written consent and in compliance with the University of the University of Pittsburgh Institutional Review Board IRB#: REN17120030/PRO14010101.

## Author Contributions

SG, NR, SP, and AL conducted the experimental design. SG and NR performed patient/sample collection. SP and MB performed data analysis with the guidance of DA. MB wrote the original draft of the manuscript. All authors reviewed and helped edit the manuscript.

## Funding

There was no commercial affiliation. Funds were provided by the National Institutes of Health grant for the University of Pittsburgh Pepper Older Americans Independence Center (P30AG024827) and by discretionary monies from the Office of the Senior Vice Chancellor for the Health Sciences, University of Pittsburgh, a non-profit entity. Neither funding source played any role in the study design, data collection and analysis, decision to publish, or preparation of the manuscript.

## Conflict of Interest

The authors declare that the research was conducted in the absence of any commercial or financial relationships that could be construed as a potential conflict of interest.

## Abbreviations

SVM, support vector machine; TFBS, transcription factor binding site; BMI, body mass index; CADD, Combined Annotation Dependent Depletion; SIFT, sorting intolerant from tolerant; GWAS, genome wide association study; ROC, receiver operating characteristic; AUC, area under the curve.
