# Supplementary material for: Exonic Variants in Aging-Related Genes Are Predictive of Phenotypic Aging Status
Source: Front Genet. 2019 Dec 19;10:1277. doi: 10.3389/fgene.2019.01277 (PMC6931058; doi:10.3389/fgene.2019.01277)
Supplement: Supplementary file 1 [file DataSheet_1.pdf]

This file includes nine figures (figure S1-S9) and six tables (table S10-S15).

Data set	Number of SNPs
Initial Wellderly	8018
Initial UPM	25273
Final (both)	5896

S1 Table. Number of variants before and after QC in both cohorts.

Subset	Number of Variants
Variants	5896
Rare Variants	2773
Very Rare Variants	998
Medium CADD Score Variants	530
High CADD Score Variants	140
Target Variants	1962
Rare Target Variants	963
Very Rare Target Variants	331
Medium CADD Score Target Variants	203
High CADD Score Target Variants	60
Variants +/- 50kb of Target Genes	4522
Rare Variants +/- 50kb of Target Genes	2102
Very Rare Variants +/- 50kb of Target Genes	751
Medium CADD Score Variants +/- 50kb of Target Genes	410
High CADD Score Variants +/- 50kb of Target Genes	113
Exon Variants	228
Rare Exon Variants	99
Very Rare Exon Variants	45
Medium CADD Score Exon Variants	51

High CADD Score Exon Variants	20
CodingAnnoType_variants	162
Known Variants	5067
Unknown Variants	829
TFBS Variants	1180
SIFT Deleterious Variants	28
SIFT Tolerant Variants	56
eQTL Variants	30
GWAS Variants	1540
Control	5896

S2 Table. Number of variants in each data set for Random Forest predictive modeling.

Chromosome	Position	rsID	Gene	P-Value	Adj. P-Value	tAF
1	156079083	rs915180	LMNA	0.0015	1	0.56
1	156078249	rs915179	LMNA	0.0017	1	0.56
8	30910690	rs6989940	WRN	0.0017	1	0.07
8	30911082	rs6991755	WRN	0.0017	1	0.07
1	156045662	rs10047112	MEX3A	0.0028	1	0.59
1	155993678	rs55935614	SSR2	0.003	1	0.05
1	156074845	rs6661281	LMNA	0.0035	1	0.6
8	30909416	rs55932348	WRN	0.0037	1	0.04
8	30897476	rs55895301	WRN	0.0039	1	0.05
8	30907657	rs56111434	WRN	0.0039	1	0.06
8	30926637	rs11574211	WRN	0.0044	1	0.06
8	31026051	rs2553257	WRN	0.0045	1	0.9
9	21999800	rs3218007	RP11-149I24	0.0057	1	0.16
9	22000247	rs3218005	RP11-149I24	0.0057	1	0.16
9	22000841	rs3218002	RP11-149I24	0.0057	1	0.16
9	22053895	rs17756311	CDKN2B-AS1	0.0059	1	0.07
9	22054164	rs74655961	CDKN2B-AS1	0.0059	1	0.07

9	22054356	rs17694572	CDKN2B-AS1	0.0059	1	0.07
8	30993804	rs56359757	WRN	0.0067	1	0.04
8	31018962	rs67722242	WRN	0.0067	1	0.04

S3 Table. Single Variant Association of the aging phenotype.

Label	Subset	Number of Variants
1	Variants	5896
2	Rare Variants	2773
3	Very Rare Variants	998
4	Medium CADD Score Variants	530
5	High CADD Score Variants	140
6	Target Variants	1962
7	Rare Target Variants	963
8	Very Rare Target Variants	331
9	Medium CADD Score Target Variants	203
10	High CADD Score Target Variants	60
11	Variants +/- 50kb of Target Genes	4522
12	Rare Variants +/- 50kb of Target Genes	2102
13	Very Rare Variants +/- 50kb of Target Genes	751
14	Medium CADD Score Variants +/- 50kb of Target Genes	410
15	High CADD Score Variants +/- 50kb of Target Genes	113
16	Exon Variants	228
17	Rare Exon Variants	99
18	Very Rare Exon Variants	45
19	Medium CADD Score Exon Variants	51
20	High CADD Score Exon Variants	20
21	CodingAnnoType_variants	162

22	Known Variants	5067
23	Unknown Variants	829
24	TFBS Variants	1180
25	SIFT Deleterious Variants	28
26	SIFT Tolerant Variants	56
27	eQTL Variants	30
28	GWAS Variants	1540
29	Control	5896

S4 Table. Labels for Random Forest Models based on data filter.

Chrom.	Position	Reference Allele	Alternate Allele	Gene	rsID	tAF	Exon	SIFT Category	CADD Score	GINI Score
1	226555302	A	G	PARP1	rs1136410	0.24	17/23	tolerated	20.9	1.262392214
13	103528002	G	C	ERCC5	rs17655	0.38	15/15	deleterious	18.44	1.150069968
1	156099669	T	G	LMNA	rs513043	0.17	2/13	deleterious	18.33	1.027403841
16	14029033	G	A	ERCC4	rs1800067	0.03	8/11	deleterious	36	0.81013859
15	91354521	G	A	BLM	rs7167216	0.07	19/20	deleterious	15.2	0.445981079
15	91326099	C	T	BLM	rs11852361	0.05	13/20	deleterious	19.45	0.406167566
9	32974493	C	T	APTX	rs104894103	NA	7/8	NA	35	0.315615679
10	50690821	G	A	ERCC6	rs114852424	NA	4/15	deleterious	35	0.269875268
15	89873364	C	G	POLG	rs61752784	0.0037	3/23	deleterious	32	0.24331742
15	89866691	C	G	POLG	rs121918054	5.00E-04	1/4	deleterious	25.5	0.228743149
8	31012237	C	G	WRN	rs78488552	9.00E-04	32/35	deleterious	19.22	0.222321772
9	32984657	A	T	APTX	rs141195622	0.0014	6/8	tolerated	18.05	0.217668298
8	31030535	C	T	WRN	rs11574410	0.0018	35/35	NA	40	0.21050682
13	103518693	G	A	ERCC5	rs142438319	NA	10/15	deleterious	22.5	0.203230472
10	50680422	C	T	ERCC6	rs145720191	0.0023	10/15	deleterious	32	0.189907287

10	50708599	C	T	ERCC6	rs41549213	NA	7/21	tolerated	21.9	0.166872695
13	103527849	G	C	ERCC5	rs9514066	1	15/15	deleterious	17.11	0.108443334
10	50732202	T	G	RP11-123B3.6	rs4253046	NA	5/6	deleterious	17.18	0.036390003
15	89873337	T	A	POLG	rs138929605	NA	3/23	tolerated	15.41	0.014827359

S5 Table. Phenotypic information for top predictors from the best performing model based on the literature.

snp ID	Nearest Gene	GWAS P-value	PMID	Phenotype based on the literature
rs7167216	BLM	5.10E-05	23555315	Breast cancer
rs1136410	PARP1	4.20E-04	20686565	LDL cholesterol
rs1136410	PARP1	9.50E-04	20686565	Total cholesterol
rs1136410	PARP1	1.00E-03	17554300	Hypertension, combined control dataset, gender differentiated
rs1136410	PARP1	1.90E-03	20935629	Waist hip ratio
rs1136410	PARP1	2.60E-03	22504419	Infant head circumference
rs17655	ERCC5	1.40E-02	21909115	Diastolic blood pressure (DBP)
rs7167216	BLM	1.70E-02	23722424	College completion
rs11852361	BLM	2.10E-02	23722424	College completion
rs1136410	PARP1	2.10E-02	23202125	Coronary artery disease (CAD)
rs1800067	ERCC4	2.30E-02	20686565	HDL cholesterol
rs1136410	PARP1	2.30E-02	22210626	Age at death with kuru exposure
rs1136410	PARP1	2.60E-02	23474815	Refractive error
rs1136410	PARP1	3.20E-02	19060906	LDL cholesterol
rs1136410	PARP1	3.40E-02	21124317	Neuroblastoma (brain cancer)
rs11852361	BLM	3.50E-02	20339536	Triglycerides change with statins

rs7167216	BLM	3.80E-02	20339536	Triglycerides change with statins
rs7167216	BLM	3.90E-02	20339536	Total cholesterol change with statins
rs1136410	PARP1	4.00E-02	22837397	Salmonella-induced pyroptosis
rs1136410	PARP1	4.10E-02	18483556	Eye color
rs1136410	PARP1	4.20E-02	20383146	Chronic kidney disease

S6 Table. Information for GWAS variants in the top performing model.

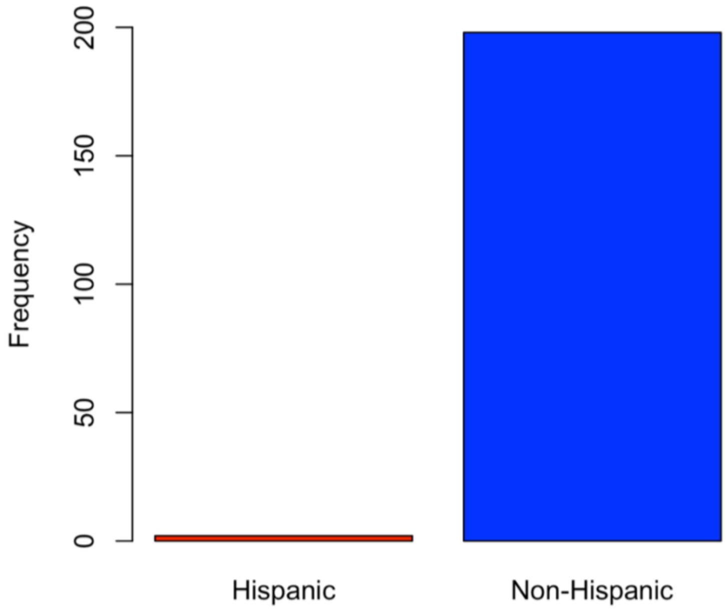

S1 Fig. Bar plot of the discovery cohort Hispanic background distribution.

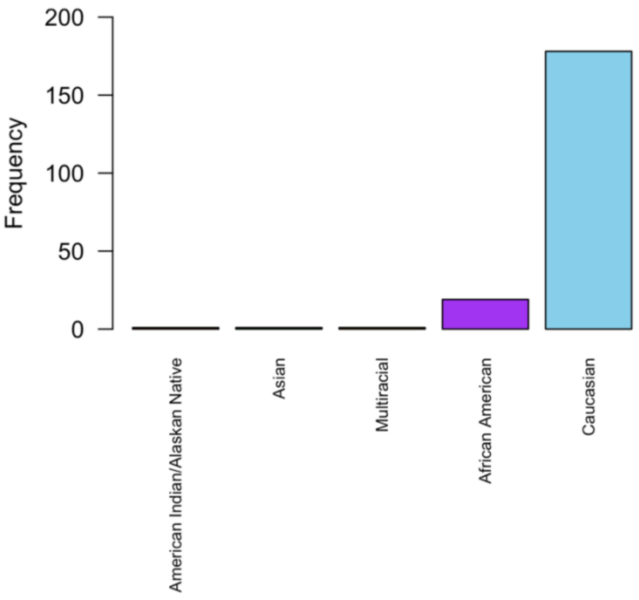

S2 Fig. Bar plots of the non-Hispanic discovery cohort ethnic background.

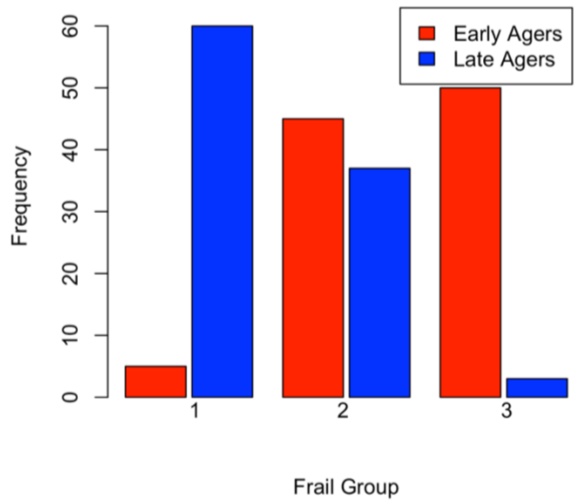

S3 Fig. Bar plot of discovery cohort frailty group (1-3) by age group (Early and Late).

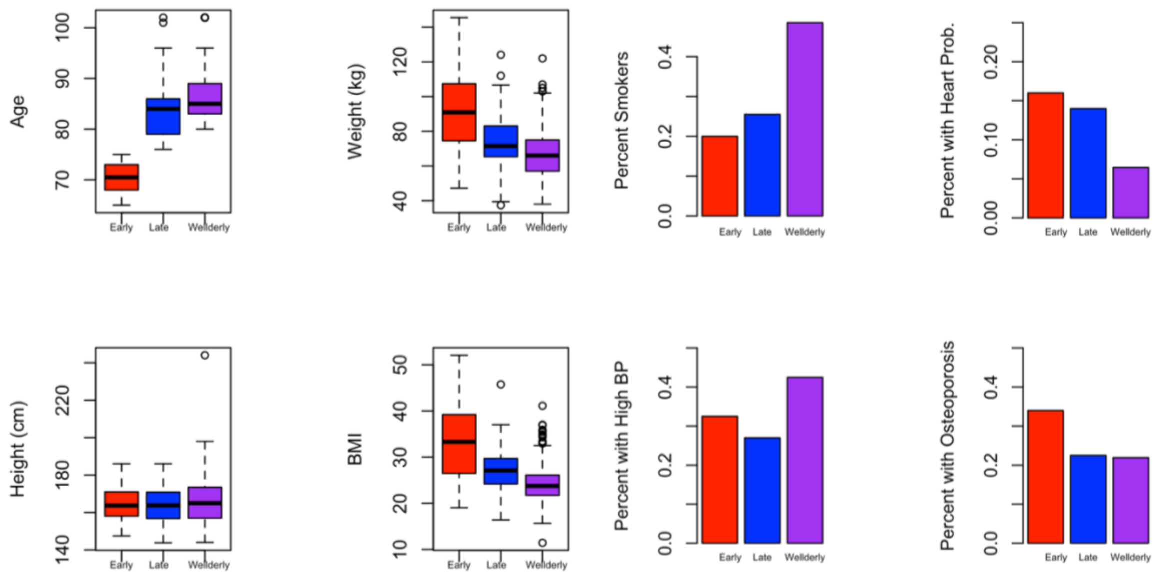

S4 Fig. Box and bar plots of overlapping phenotype data in the training and discovery cohorts.

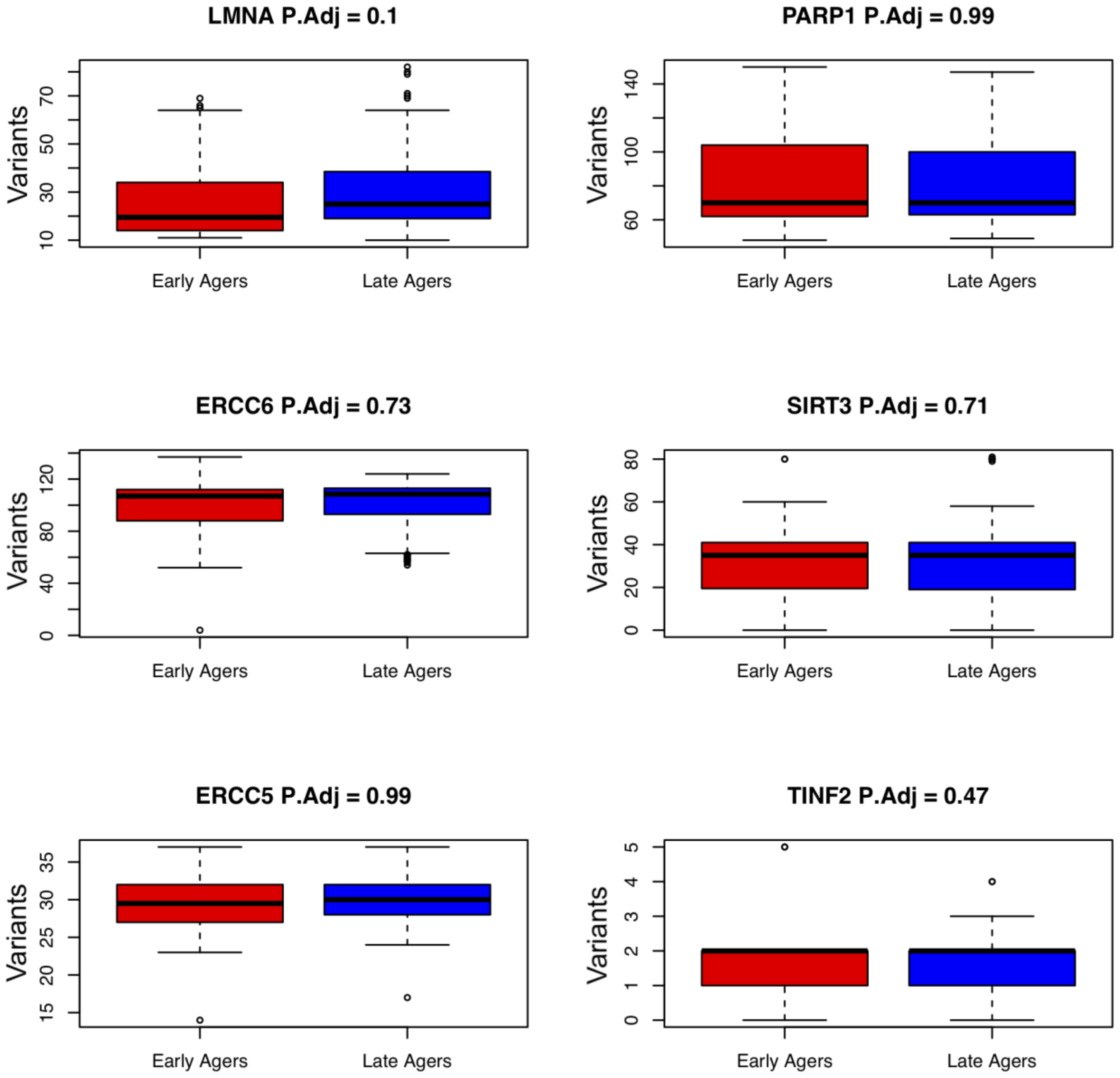
LMNA P.Adj = 0.1
PARP1 P.Adj = 0.99
ERCC6 P.Adj = 0.73
SIRT3 P.Adj = 0.71
ERCC5 P.Adj = 0.99
TINF2 P.Adj = 0.47
Variants
Early Agers
Late Agers
10
30
50
70
60
100
140
0
40
80
120
0
20
40
60
80
15
20
25
30
35
0
1
2
3
4
5

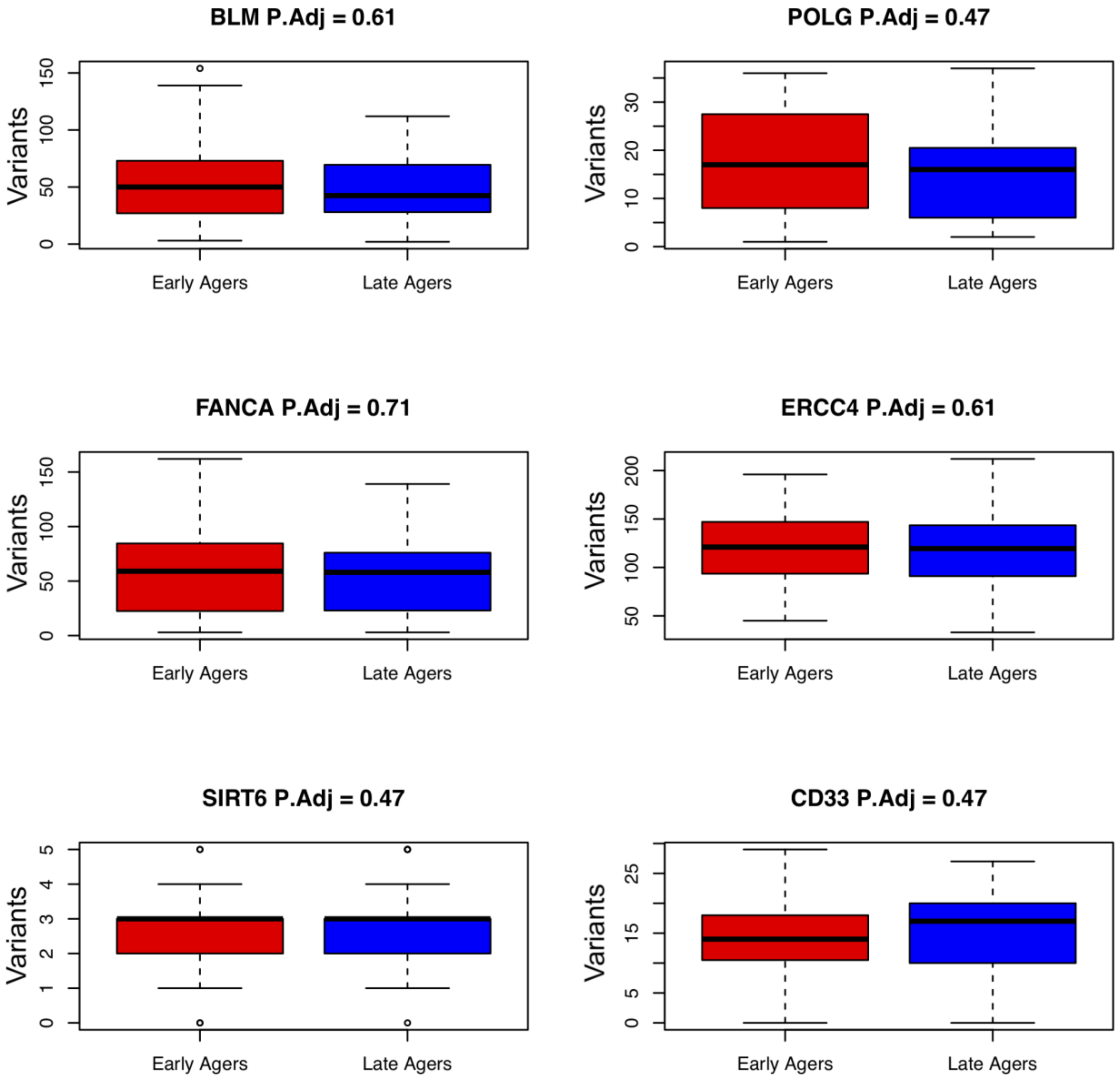
BLM P.Adj = 0.61
POLG P.Adj = 0.47
FANCA P.Adj = 0.71
ERCC4 P.Adj = 0.61
SIRT6 P.Adj = 0.47
CD33 P.Adj = 0.47
Variants
Early Agers
Late Agers

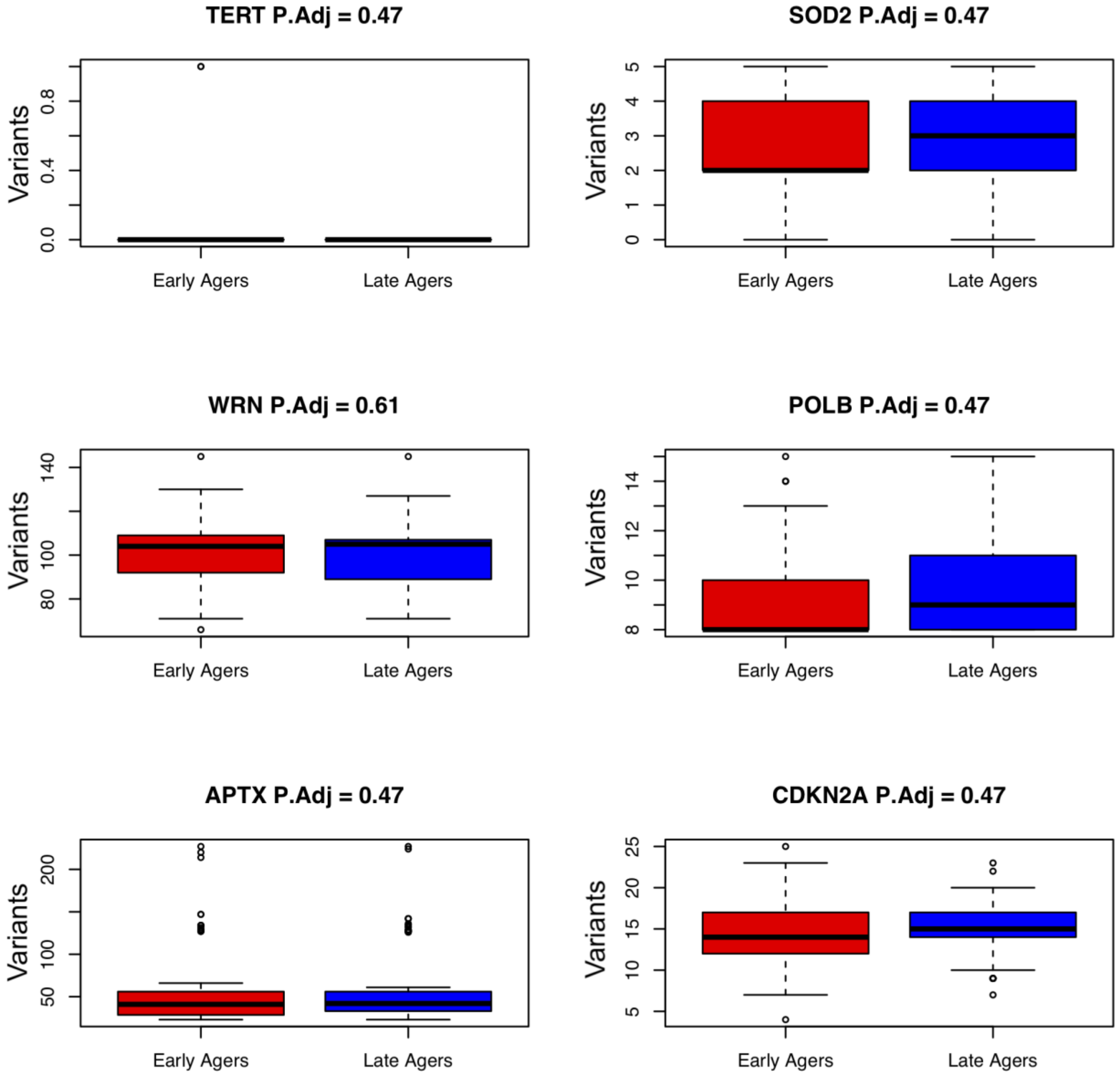

S5 Fig. Box plots on non-reference allele counts in each target gene by age group.

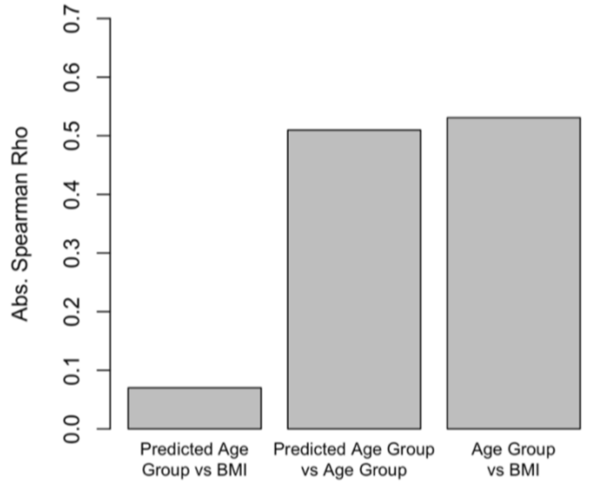

S6 Fig. Bar plot of the Spearman Rho correlation coefficient between the predicted age group and BMI, the predicted age group and the actual age group, and the actual age group and BMI.

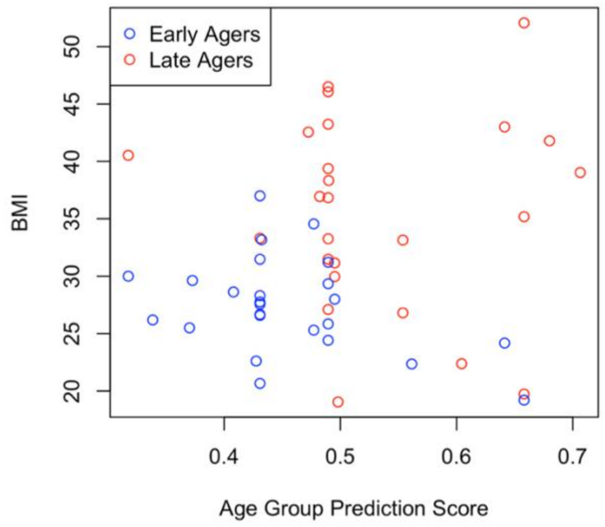

S7 Fig. Scatter Plot of the age group prediction versus BMI for the discovery and validation cohorts in the top model.

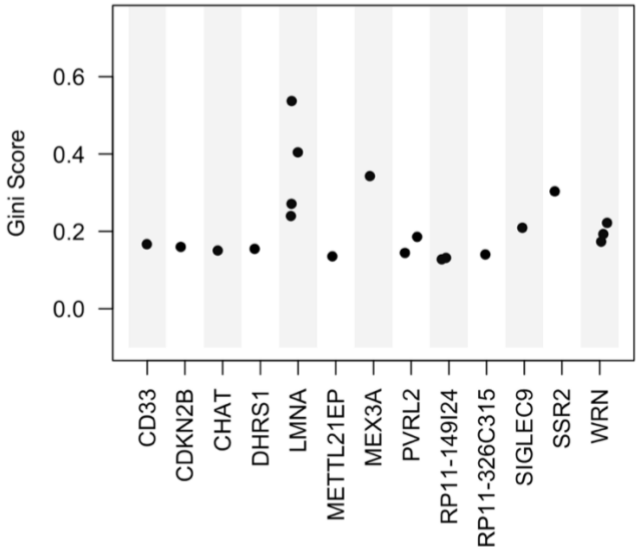

S8 Fig. Scatter Plot of the mean Gini Scores for each variant by gene from the non-filtered model.

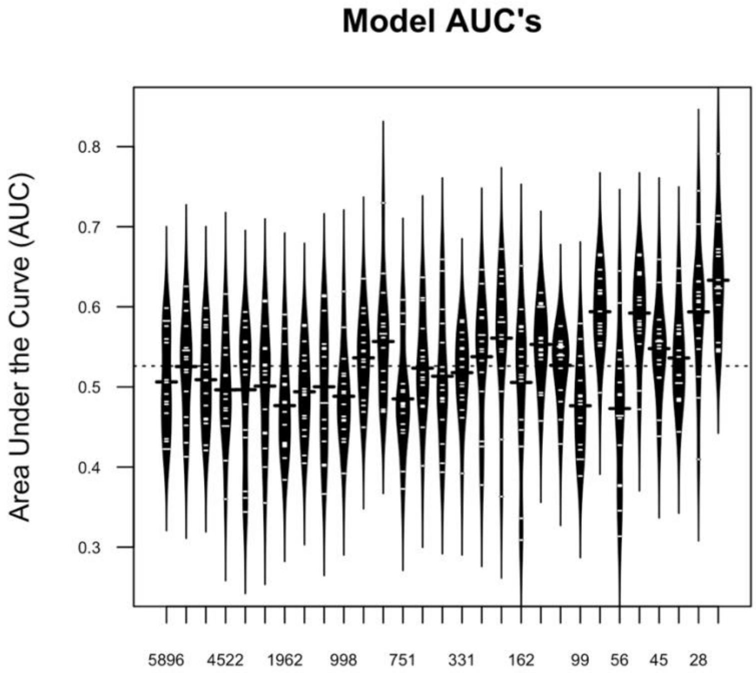

S9 Fig. Bean plot of random forest model AUCs
